# Rapid Evolution of Metastases in Patients with Treated G3 Neuroendocrine Tumors Associated with NEC-Like Transformation and *TP53* Mutation

**DOI:** 10.1007/s12022-024-09827-y

**Published:** 2024-10-09

**Authors:** Atsuko Kasajima, Nicole Pfarr, Eva-Maria Mayr, Ayako Ura, Elisa Moser, Alexander von Werder, Abbas Agaimy, Marianne Pavel, Günter Klöppel

**Affiliations:** 1 Department of Pathology, Technical University Munich, TUM School of Medicine and Health, Munich, Germany; 2 Department of Internal Medicine II, Technical University Munich, TUM School of Medicine and Health, Munich, Germany; 3 Department of Pathology, University Hospital Erlangen (UKER), Erlangen, Germany; 4 Internal Medicine, University Hospital Erlangen (UKER), Erlangen, Germany; 5 Comprehensive Cancer Center Erlangen-EMN (CCC ER-EMN), Friedrich-Alexander University Erlangen-Nürnberg (FAU), Erlangen, Germany

**Keywords:** Neuroendocrine tumors, Progression, Transformation, NEC-like NET G3

## Abstract

Little is known about the morphomolecular features of G3 neuroendocrine tumors (G3NETs) under prolonged systemic treatments, although rapid progression is increasingly observed. This longitudinal study aims to elucidate the course and morphomolecular features of metastasized G3NETs with high-grade transformation. Clinical and histological findings in 40 patients with metastasized and treated G3NETs, which were histologically examined at least twice with an interval time of more than 6 months (median 27), were reviewed and the morphomolecular changes recorded and assigned to treatment. Neuroendocrine carcinoma (NEC)-like histology defined by high-grade atypia, diffuse growth pattern, and/or necrosis was identified in nine (22%) G3NETs (seven pancreatic, two rectal) patients. All NEC-like tumors showed a significantly higher Ki67 increase and longer interval time between first and last examination than non-NEC-like G3NETs (53 vs. 19% and 60 vs. 24 months, respectively). Moreover, all NEC-like G3NETs had *TP53* (100%), but rarely *RB1* (12%) mutations, and retained NET-typical mutations such as *MEN1* or *DAXX *(five of the pancreatic NETs). The last treatments received prior to the NEC-like transformation included PRRT (*n* = 3), somatostatin analog, everolimus, sunitinib (*n* = 1 each), and alkylating agents (*n* = 2). Abrupt clinical progression in patients with metastasized G3NETs is associated with a significant increase in Ki67, accelerated growth, and NEC-like histology. These findings are most likely attributable to the novel *TP53* mutation, which was detected in all nine cases at the last evaluation. However, none of the cases exhibited a complete transformation to a typical NEC, as the tumors retained partial histological and genetic features of NETs.

**Supplementary Information:**

The online version contains supplementary material available at 10.1007/s12022-024-09827-y.

## Introduction

G3 neuroendocrine tumors (G3NETs) are classified as well-differentiated neuroendocrine neoplasms (NENs), though they may exhibit characteristics that overlap with those of poorly differentiated NENs, called neuroendocrine carcinomas (NECs). This is due to their proliferative activity exceeding 20% Ki67. The distinguishing features that set them apart from NECs are the morphomolecular characteristics that align with those of G1 and G2 NETs [1–3]. G3NETs retain an organoid growth pattern, which is more often solid than trabecular, and are associated with low to moderate nuclear atypia. NECs frequently manifest a diffuse growth pattern, high-grade nuclear atypia, and tumor cell necrosis. The median Ki67 value for G3NETs is 30%, whereas NECs typically exhibit Ki67 values of approximately 60% [4]. It is of particular significance that G3NETs display wild-type *TP53* and *Rb1* gene profiles, whereas NECs frequently exhibit *TP53* mutations and often present with *Rb1* gene abnormalities [1–3, 5, 6]. Furthermore, there are approximately 40% of pancreatic NETs with mutations in *DAXX/ATRX* and *MEN1*, in addition to alternative lengthening of telomeres (ALT), a phenomenon not observed in NECs [5, 7]. In terms of clinical outcomes, patients with G3 PanNETs tend to experience less favorable outcomes than those with NET G1/G2. Nevertheless, they continue to demonstrate superior survival rates in comparison to patients with NECs [8–10]. These distinctions between NET and NEC are also observed in extrapancreatic NENs, including those of gastric [11], colorectal [12], and bronchopulmonary origin [13, 14]. Both platinum/etoposide and alkylating agents have been employed in the treatment of G3NET and NEC patients. However, G3NET patients exhibit a diminished response to platinum-based chemotherapy when compared to NEC patients with a Ki67 index > 55% [15, 16]. A retrospective study of PanNEN reported response rates to platinum agents of 10% in G3NET, but 37% in NEC. However, response rates to alkylating agents were similar, with 50% in both groups. Nevertheless, G3NET patients exhibited a markedly superior overall survival compared to NEC patients [17].

The exact evolution of G3NETs remains incompletely understood. Thus far, it is evident that when Ki67 indices are compared across multiple tumor samples from individual patients, the value increases in both synchronous and metachronous metastasis of gastroenteropancreatic NETs [18–21, 22] as well as pulmonary NETs [23]. This finding correlates with poor patient outcome [20–22]. Moreover, G3NETs, but not NETs G1/G2, frequently exhibit a focal high-grade histological feature [24]. The observed increase in Ki67 and higher histological atypia during disease progression suggests that G3NETs may arise from lower grade NETs [18–22], recently reviewed in [25]. However, an evolutionary progression from G3NET to NEC was regarded as improbable on account of the evident distinctions in the biological characteristics of the two tumor entities (see above). With regard to the genetic profiling of the two entities, a limited number of studies have identified a genetic overlap between G3NETs and NECs, with a particular focus on *TP53* or *RB1* mutations [26–29]. Moreover, some authors have proposed the concept of a potential “NET to NEC” transformation, based on the observation of histological alterations over time [30] or the identification of commonly altered gene clusters in NETs and NECs [31, 32]. Additionally, it has been postulated that specific therapeutic regimens may be associated with an abrupt transformation of NETs to high-grade NENs [30].

The present studies have yet to elucidate whether a transformation from G3NET to NEC occurs, particularly in patients exhibiting rapid clinical progression. Additionally, the number of NETs that undergo this transition, the time frame in which it may occur, and the potential association with previous therapies remain unclear. The objective of this study was to elucidate the progression characteristics in a series of metastasized G3NETs from patients who received systemic treatment and had tissue samples taken at the time of diagnosis and later in the course of the disease.

## Materials and Methods

### Tissue and Data Assembling

Formalin-fixed, paraffin-embedded NET tissue blocks from 1000 consecutive patients were retrieved from the files of the Department of Pathology and the Consultation Centre for Pancreatic and Endocrine Neoplasms, Technical University of Munich, TUM School of Medicine and Health, Germany, between April 2009 and June 2024. The consultation series was mostly the same as previously used to analyze the immunohistochemical and clinical features of G3NET [1] and mesenchymal neoplasms with neuroendocrine features mimicking NETs or neuroendocrine carcinomas (NECs) [33]. Samples from 115 patients were examined histologically at least twice. Nine patients with multifocal type 1 gastric NETs were excluded in order to retain NET patients with unifocal tumor origin (unifocal NET in Supplementary Fig. 1). Also excluded were 44 patients who had repeated examination within 6 months (Supplementary Fig. 1).The remaining 62 patients had metastatic NET at the time of the last examination. In these patients, repeated histological examination was most commonly required to verify the diagnosis including tumor entity, Ki67 index, and/or identification of therapy-related molecular features (*n* = 52, 84%). In addition, eight patients (13%) underwent tumor debulking after recurrence of metastatic disease and two patients (3%) underwent resection of the primary after conducting systemic therapy based on initial histological evaluation. If more than two samples were available, only the first and the last tissue samples were included in the analysis. Patient age, sex, tumor origin, tissue examined (biopsy or resected specimen), changes in WHO grade, and differences between the Ki67 indices of the two examinations (called delta Ki67) are shown in Supplementary Table 1. Data regarding systemic treatments (peptide receptor radionuclide therapy (PRRT) targeting the somatostatin receptor, chemotherapy, among others) or nonsurgical ablative or locoregional treatments (e.g., radiotherapy, selective internal radiation therapy (SIRT)) administered between the first and the last visit were available in 31 of 40 G3NET patients. The last treatment prior to the last histological examination was included for comparative analysis. Survival data of the patients regarding progression free survival (PFS) and disease-specific survival (DSS) were extracted from the medical record. This study received IRB approval of the local ethic committee 2022–396-DFG-SR, TUM School of Medicine, Munich, Germany.

### Histopathological Evaluation

All tissue samples were reviewed by two experts in endocrine and pancreatic pathology (AK and GK). The final diagnosis was based on the current WHO classifications of tumors of the endocrine organs [34]. All well-differentiated NENs, regardless of their origin, were classified and graded as NETs [34]. NEC-like features were defined according to three selected and slightly modified histological criteria proposed by Elvebakken: (1) confluent pattern without regular vascularization, (2) high nuclear variation with pleomorphism, and (3) presence of geographic necrosis [15]. The difference in the Ki67 index between the first and the last examination was defined as “delta Ki67 index” (see above).

### Immunohistochemical Evaluation

Immunohistochemical staining was performed on 2-µm sections using an automated system (Benchmark XT; Ventana/Roche, AZ, USA). Details of immunohistochemical staining are shown in Supplementary Table 2. Nuclear Ki67 labelling was counted in more than 500 tumor cells in highest density (hot spot) and its percentage was expressed as Ki67 index (%). Membranous expression of the somatostatin receptor 2 (SST2) was evaluated according to the previously described method, and a score of 2 + and a score of 3 + were considered positive [13]. Abnormal p53 expression was defined as strong nuclear immunoreactivity in more than 20% of tumor cells or as complete absence of expression [3]. Rb1 nuclear expression in less than 10% of tumor cells was defined as loss/abnormal expression [13]. Immunohistochemical staining of p53 and Rb1 was performed in both initial and last examinations in 29 and 27 patients, respectively (Table 1).

**Table 1:** Clinicopathological features of G3 neuroendocrine tumor patients histologically examined twice due to tumor progression

		Total	NET G3		*p*-value
			Without NEC-like features	With NEC-like features	
Total *N* (%)		40 (100)	31 (78)	9 (22)	
Age	Median (range)	58 (33–81)	61 (33–81)	51 (38–71)	NS
Sex	Male to female	20:20	13:16	7:2	NS (0.07)
Primary organ	*N* (%)				
	Pancreas	22 (55)	15 (48)	7 (78)	0.01
	Lung	10 (25)	10 (32)	0	
	Ileum	3 (8)	3 (10)	0	
	Rectum	2 (5)	0	2 (22)	
	Others	3 (8)	3 (19)^e^	0	
Functionality	Non-functional	34 (85)	27 (87)	7 (78)	NS
	Functional	6 (15)	4 (14)^g^	2 (22)^h^	
Initial Grade	G1	4 (10)	4 (13)	0	NS
	G2	24 (60)	17 (55)	7 (78)	
	G3	12 (30)	10 (32)	2 (22)	
Delta Ki67	Median (range)	25 (1–68)	19 (1–42)	53 (37–68)	< 0.0001
Initial Ki67 index	Median (range)	12 (1–40)	15 (1–40)	10 (3–28)	NS
Last Ki67 index	Median (range)	40 (15–85)	40 (15–60)	65 (50–85)	< 0.0001
Interval time (months)	Median (range)	29 (7–180)	24 (7–180)	60 (30–168)	< 0.01
P53 status^a^	N (%)				
Initial-last	Normal–normal	27 (73)	27 (96)	0	<0.0001
	Normal–abnormal	9 (24)	1 (4)	8 (89)	
	Abnormal–abnormal	1 (3)	0	1 (11)	
RB1 status^b^	*N* (%)				
Initial–last	Normal–normal	33 (94)	25 (96)	8 (88)	NS
	Normal–abnormal	2 (6)	1 (4)	1 (13)	
SST status^c^	*N* (%)				
Initial–last	Negative–negative	10 (26)	9 (31)	1 (11)	NS
	Positive–positive	28 (74)	20 (69)	8 (89)	
Disease-specific survival	3-year survival rate (%)	93	95	88	NS
	5-year survival rate (%)	89	90	66	

*NET* neuroendocrine tumor, *NEC* neuroendocrine carcinoma, *SST* somatostatin receptor, data missing in a) 11 cases, b) 13 cases, c) 8 cases, d) 1 duodenal, 2 renal, e) 1 gastric, breast, presacral each, f) 1 Cushing syndrome and Zollinger-Ellison syndrome (ZES), g) 1 glucagonoma, carcinoid syndrome, ZES and Cushing syndrome each, h) 1 hyperglucagonemia, insulinoma each

### Molecular Genetic Studies

A molecular genetic analysis was performed on 16 G3NET patients (40%) using tumor tissue obtained from the last examination. Comparable data from the tissue at initial visit was available in three cases. As previously described, genetic analysis was conducted using the TruSight Oncology 500 assay (Illumina, San Diego, CA, USA), in accordance to the manufacturer’s protocol [35].

### Statistical Analyses

JMP Pro version 17.0.0 software (SAS Institute, Inc., Cary, NC, USA) was used for all statistical analyses. A correlation coefficient was calculated using Spearman’s method. Pearson’s chi-squared test or Fisher´s exact test was used to compare sample sizes between groups. The Wilcoxon test was used to compare continuous values or scores between multiple groups that were not normally distributed according to the Shapiro–Wilk test. The probability of differences in PFS and DSS was determined using the Kaplan–Meier method, with a log-rank test for significance. A *p*-value of < 0.05 was considered statistically significant.

## Results

### G3NET Features

**Fig. 1:**
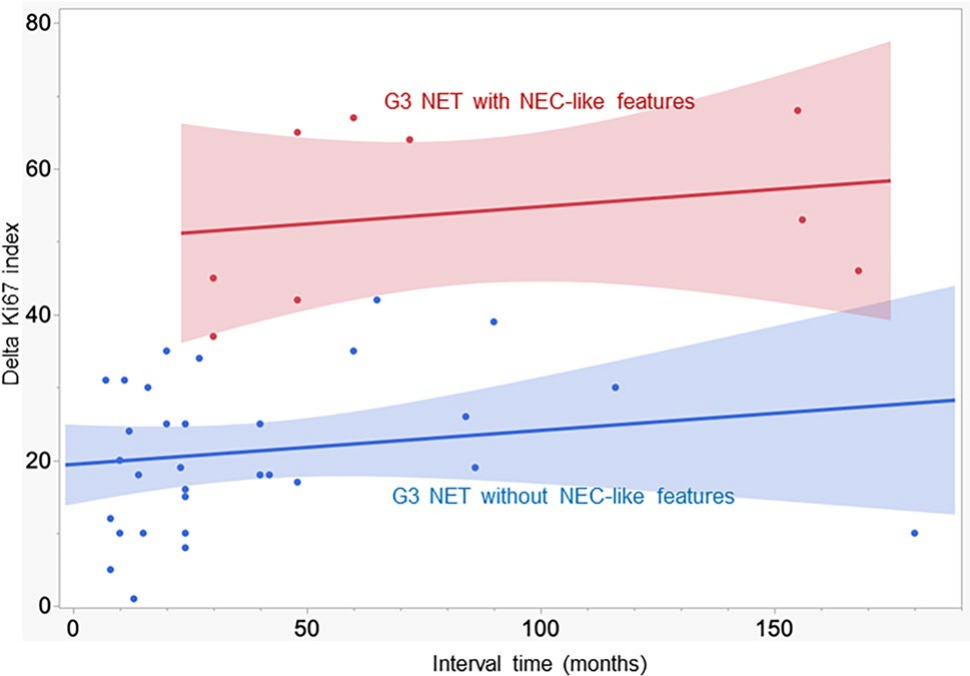
Graph illustrating the individual delta Ki67 index (Y-axis) related to the interval time (X-axis) between initial and last examinations of 9 G3 neuroendocrine tumors (G3NETs) with NEC-like features (red) compared to 31 G3NETs without NEC-like features (blue)

At the time of the last examination, 40 out of 62 patients (65%) had been diagnosed with G3NET (the median interval time 29 months, range from 7 to 180 months). The remaining 22 (35%) patients were diagnosed with G1NET or G2NET at the time of the last examination (Supplementary Fig. 1, Supplementary Table 1). Of the G3NET patients, 4/40 (10%) and 24/40 (60%) were diagnosed with G1NET and G2NET, respectively, at the initial visit (Table 1). The remaining 12 patients (30%) exhibited a G3NET in both samples (Table 1). In all cases, the Ki67 index was found to be elevated at the last examination, with a median delta Ki67 value of 25. A positive correlation was observed between higher delta Ki67 values and longer interval times (*p* = 0.002, Fig. 1). The majority of G3NETs were located in the pancreas (22/40, 55%), followed by the lung (10/40, 25%), ileum (3/40, 8%), and rectum (2/40, 5%). Additionally, rare sites included the stomach, presacral region, and breast (Table 1).

### Clinicopathological Findings in NEC-Like G3NET

Nine out of 40 G3NETs (22%) showed NEC-like features at the last examination. There was no difference in age between G3NET patients with and without NEC-like features. The frequency of G3NETs with NEC-like transformation was higher in male patients (78% of cases) than in female patients, although this difference was not statistically significant (*p* = 0.07). Most (7/9, 78%) of the NEC-like G3NETs originated from the pancreas (six from the tail), and two from the rectum (Table 1). The median interval between the two examinations was 60 months (range: 30 to 168), which was significantly longer than observed in G3NET patients without NEC-like features (24 months, *p* < 0.01, Table 1). None of the patients had a hereditary genetic syndrome such as MEN1 or NF1. Seven NEC-like G3NETs were non-functional. One of the other two patients developed hyperglucagonemia (max. 3460 pg/ml) in the absence of glucagonoma syndrome and with a slight decrease in serum glucagon following treatment with everolimus (ID2 in Table 2). The other patient initially presented with Zollinger-Ellison syndrome (ZES), followed by hypoglycemia (ID3 in Table 2). At the last examination, 50% of the tumor cells expressed gastrin and 5% insulin. All NEC-like G3NET patients developed rapid tumor evolution with significant enlargement of liver metastases and/or occurrence of new lesions in a short period of time, along with deterioration of the clinical condition, an elevation of serum transaminases and increase of circulating tumor markers such as chromograninA and/or NSE. In contrast, non-NEC-like G3NET patients had a gradualprogression. Three out of nine (33%) NEC-like G3NET patients and 4/31 (13%) non-NEC-like G3NET patients died of disease. There was no statistical difference in outcome (PFS and DSS) between the two groups (Tables 1 and 2).

**Table 2:** Clinicopathological and molecular features of nine patients with NET G3 with NEC-like features

ID	Age Sex	Primary	Functionality	Interval time (mon)	Delta Ki67^a^ Initial–last Ki67	NGS in last examination									Therapeutic modules used between initial and last examination	
						*TP53*	*RB1*	*MEN1*	*DAXX*	*ATRX*	Other mutations	Other findings	TMB (mut/mb)	Microsatellite status		Outcome
1	45 F	Pan tail	NF	30	37 28–65	c.413C > A (cl.4) AF: 81%	WT	WT	WT	WT	none	Amp. *FGFR3, CDKN2A, NOTCH1, TRAF2, AKT1, MAP2K2, NOTCH3, JAK3, CIC,* del. NRAS, NOTCH2, MSH2, FANCL, *IDH1, BARD1, PIK3CB, ATR, PIK3C*A ect	10.2	Stable	FOLFOX—CAP/TEM—FOLFOX—SIRT	50 months progress
2	71 M	Pan tail	Hyperglucagonemia	72	64 21–85	c.832C > A (cl. 5) AF: 95%	WT	WT	WT	WT	none	amp. *MYC, BBX::MYC*	0.8	Stable	STZ/5Fu—CAP/TEM—SSA—EVE	36 months disease-related death
3	51 M	Pan tail	ZES followed by hypoglycemia	48	42 10–52	c.824G > T (cl. 4) AF: 68%	WT	WT	c.208-1G > T (cl. 4)	WT	*mTOR* c7238G > T, c3266G > T, *SETD2* c4221del, *PBRM1* c.2576C > G	del. DNMT3A	13.4	Stable	PRRT—STZ/5FU—TKI—SIRT—RTx	53 months progress
4	38 M	Pan head	NF	60	67 3–70	c.830G > T (cl. 4) AF: 50%	WT	c.1525delC (cl. 5)	WT	WT	none	*CDKN1A* c.3_25del, *MUTYH* c.640C > T	357.1	Stable	SSA—STZ/5FU—PRRT—SSA—FOLFOX—Larotrectinib—PRRT—CPI—PRRT	93 months disease-related death
5	43 M	Pan tail	NF	30	45 5–50	c.772G > A (cl. 4) AF: 63%	WT	c.974 C > T (cl. 5)	WT	WT	*TSC2* c.1060C > T, *MSH3* c.1141delA, *BRCA2 (*splicing) c.9117 + 1G > A, *BCOR* (splicing) c.4741 + 1G > AS	Del. *CDKN2A, CDKN2B*	91.8	Stable	STZ/5FU—PRRT—CAPOX—SIRT	27 months progress
6	61 M	Pan tail	NF	48	65 10–75	c.331_332del (cl. 4) AF: 72%	WT	c.1594 C > T (cl. 4)	WT	WT	TGFBR2 c.383dup		6.3	Stable	PRRT—SSA—PRRT -CIS/ET	56 months progress
7	39 F	Pan tail	NF	156	43 12–65	c.1146del (cl. 4) AF: 40%	WT	c.1200 + 2del Splice Donor	c1560_1561delTT	WT	*BRCA1* c.4689C > G, *EPHA3* c.2584C > T	amp *FGFR1, FGF1, JAK2, PDGFRA, KRAS, KIT, KRG, FGF9, CDK6, FGF5, BRCA2, MET, ERBB3, MDM2, CDK4*	14,4	Stable	PRRT	154 months progress
8	57 M	Rec	NF	155	68 12–80	c.396G > C (cl. 5) AF: 12%	c.233 G > A	WT	WT	WT	*MAP3K1 c.3644delA*	amp. *ZNF703, GPR124, FGFR1, NCOR1, FLCN*	7.1	Stable	SSA -PRRT	168 months disease-related death
9	70 M	Rec	NF	176	40 10–50	c.388C > G (cl. 4) AF: 86%	WT	WT	WT	WT	none	Amp. *TNFRSF14, PIK3CD, RECQL4, RPS6KA4, CCND1* *del. STAG1, PIK3CB, ATR, PIK3CA, JAK2, CD274, PTEN, ATM, BRCA2*	1.6	Stable	unknown	185 months progress

*NEC* neuroendocrine carcinoma, *F* female, *M* male, *Pan* pancreas, *Rec* rectum, *NF* nonfunctional, *ZES* Zollinger-Ellison syndrome, *NGS* next generation sequencing, *cl.* class, *AF* allele frequency, *WT* wildtype, *PRRT* peptide receptor radionuclide therapy targeting somatostatin receptor type 2, *SSA* somatostatin analogue, *STZ/5FU* streptozotocin and 5-fluorouracil, *FOLFOX* folinic acid fluorouracil and oxaliplatin, *CAP/TEM* capecitabine and temozolomide, *TKI* tyrosine kinase inhibitor, *SIRT* selective internal radiation therapy

^a^Difference between initial and last Ki67 value

**Fig. 2:**
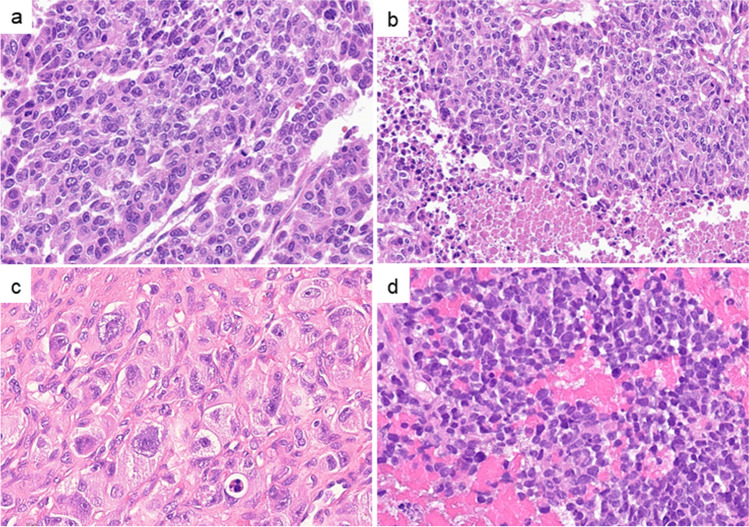
Histological features of NEC-like G3 neuroendocrine tumors. **a** Confluent growth pattern. **b** Geographic necrosis (bottom). **c** Large tumor cells with high-grad nuclear atypia and (**d**) small tumor cells resembling small cell carcinoma

**Fig. 3:**
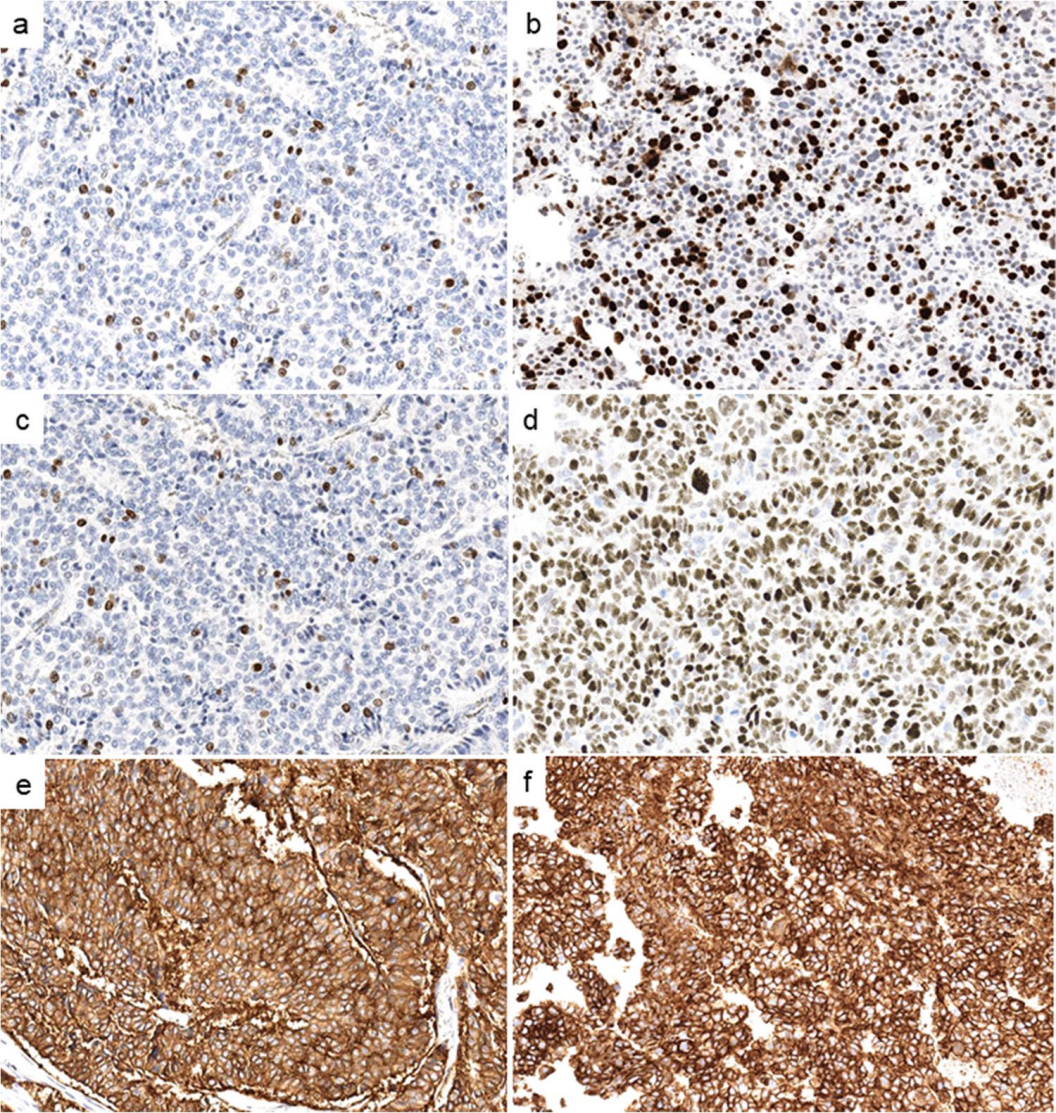
Immunohistochemical staining of NEC-like G3 neuroendocrine tumors at the initial (**a**, **c**, **e**) and last (**b**, **d**, **f**) examinations. **a** Ki67 index increased from 5% to (**b**) 65%. **c** Wild-type expression pattern of p53 changed to (**d**) overexpression. Strong expression of somatostatin receptor 2A observed in both (**e**) and (**f**)

Histologically, eight NEC-like G3NETs resembled large cell NEC (Fig. 2a, b, and c) with confluent pattern (Fig. 2a) and high nuclear variation with pleomorphism (Fig. 2c). Necrosis was observed in 8/9 (89%, Fig. 2b). One NEC-like G3NET resembled small cell NEC (Fig. 2d, ID6 in Table 2). At the initial examination, all tumors showed a NET histology, and no statistical difference in the Ki67 index was observed between G3NETs with and without NEC-like transformation (Table 1). The median Ki67 index of NEC-like G3NETs at initial and last examination was 10% (range 3 to 28) and 65% (range 50 to 85), respectively, with a median delta Ki67 value of 53 (range 37 to 68), which was significantly higher than that of G3NETs without NEC-like features (median 19, *p* < 0.0001, Table 1, Figs. 1 and 3a and b).

**Fig. 4:**
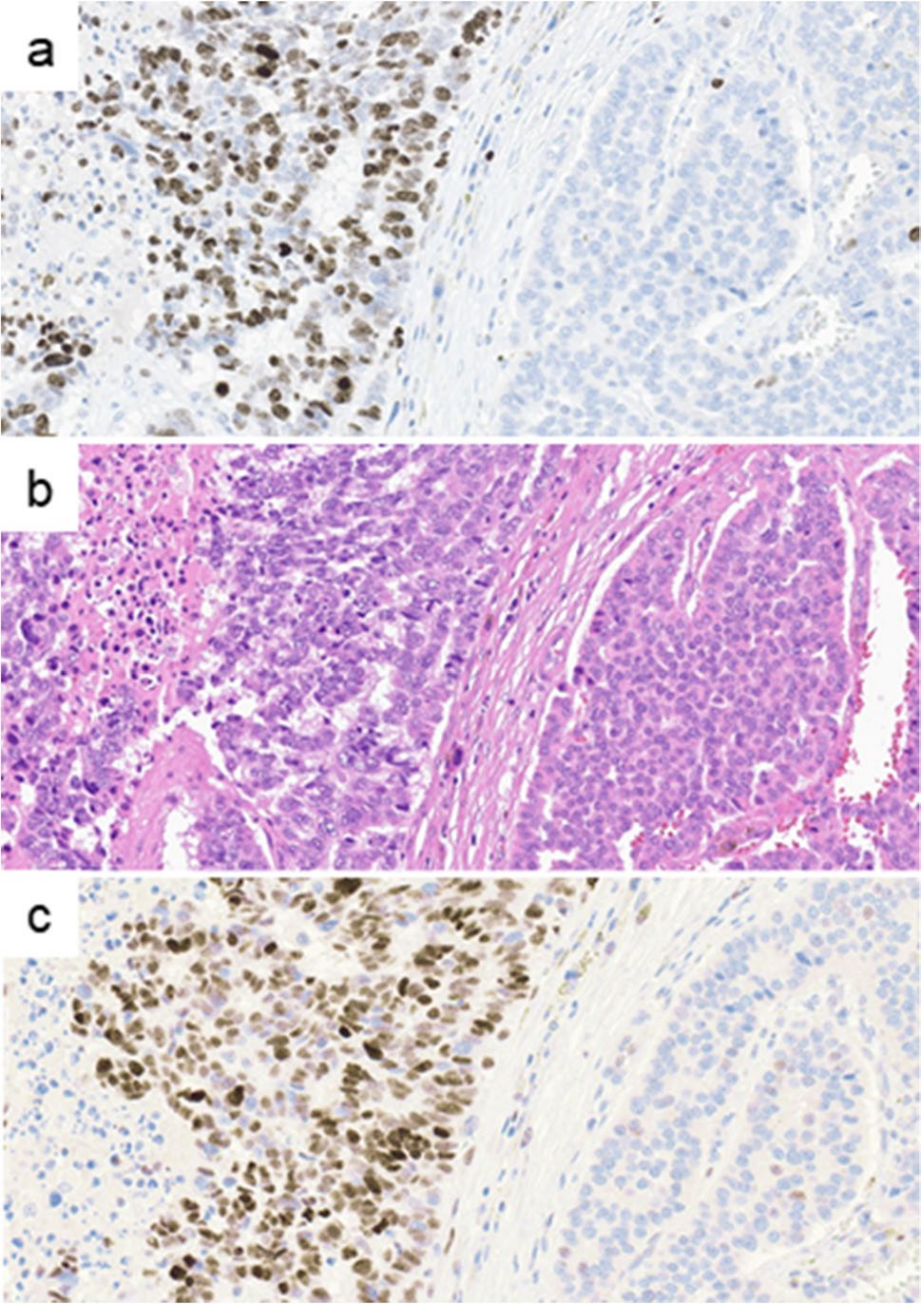
**a** Immunohistochemical staining for Ki67, **b** histological image, and (**c**) p53 staining of NEC-like G3 neuroendocrine tumor (G3NET). G3NET with an area of NEC-like features (left) showing high proliferation (**a**) and p53 overexpression (**c**) next to area of NET (right) with low proliferation (**a**) and wild-type p53 expression pattern (**c**)

Six of seven pancreatic NEC-like G3NETs corresponded to the PanNET subtypes A1 or A2 (characterized by predominantly glucagon expression) and one to subtype B (characterized by insulin expression) [36]. All NEC-like G3NETs showed an immunohistochemical p53 wild-type expression pattern at initial examination, which changed to abnormal expression in 8/9 (Fig. 3c and d). In one patient with NEC-like G3NET, p53 showed complete loss at the first examination, which was also observed at the last examination (Table 1). In contrast, G3NETs without NEC-like features retained their p53 wild-type pattern at both examinations (Table 1). Rb1 status changed from normal to abnormal expression (loss of expression) in 1/9 (13%) of NEC-like G3NET. SST2 expression was observed in 8/9 (89%) with unchanged expression status at initial and last examination (Fig. 3e and f). p16 expression was examined in 4/9 NEC-like G3NETs and 9/31 G3NETs without NEC-like features and a strong expression was observed in 3/4 (75%) of NEC-like G3NETs but none in the latter group. In tumor tissues from large specimens (especially from two tumor debulking samples), the histology showedmostly NEC-like features with high proliferation (Fig. 4a and b, ID3 in Table 2) and p53 overexpression (Fig. 4c, ID3 in Table 2) next to small foci of typical NET component without abnormal p53 expression.

### Molecular Characteristics of NEC-Like G3 NETs by NGS

NGS was performed in nine and seven G3NETs with and without NEC-like features, respectively. All (9/9) NEC-like G3NETs showed a *TP53* mutation with either pathogenic (class 5) or probable pathogenic (class 4) significance (Table 2). Conversely, none of the G3NETs without NEC-like features demonstrated this mutation. Seven out of nine cases (78%) exhibited missense mutations with disparate mutation patterns, including C > A in two cases, G > T in two cases, and G > A, G > C, and C > G in one case each. The remaining two cases demonstrated gene deletion. High allele frequencies of *TP53* mutations (> 20%) were found in 8/9 NEC-like G3NETs. Only one case (ID8 in Table 2) had an allele frequency below 20% (Table 2). The debulking sample showing only partially NET (< 5%) next to much larger NEC-like area showed also a high* TP53* allele frequency (95%, ID3 in Table 2), suggesting monoclonality (Fig. 4a and b). A single NEC-like G3NET case (1/9) exhibited a *RB1* mutation (ID8). The major genetic alterations of 12 G3NETs from the pancreas (G3PanNETs), comprising seven cases with and five without NEC-like features, are presented in Supplementary Table 3. *MEN1* mutations were identified in four of the seven G3PanNETs with NEC-like features (57%) and in two of the five G3PanNETs without NEC-like features (40%). No significant differences were observed in the prevalence of *MEN1, DAXX*, or *ATRX* mutations, tumor mutation burden, microsatellite status, or PD-L1 status between NEC-like G3NETs and non-NEC-like G3NETs.

### Treatment of NET G3 Patients Prior to NEC-Like Transformation

G3NET patients with NEC-like features received a minimum of one (with an average of four) systemic treatment regimen prior to the onset of NEC-like transformation. These treatments included PRRT (*n* = 3), somatostatin analogues, everolimus, sunitinib (*n* = 1 each), and alkylating agents (*n* = 1). A similar course of treatment was administered into G3NETs that did not undergo NEC-like transformation prior to the last examination. However, patients who did not exhibit NEC-like features received a mean of two treatment lines, which was statistically significant lower number than that received by patients with NEC-like features (*p* = 0.042, Supplementary Table 4).

## Discussion

The NET and NEC are believed to represent two distinct entities with differing biological, histological, and genetic characteristics [34, 37]. The aforementioned concept has recently been challenged by observations that suggest a transformation of G3NET to NEC [30]. In this longitudinal retrospective investigation of G3NET patients with a sudden worsening of the clinical condition, we examined the progression of metastasized NETs under systemic therapy with follow-up biopsies. As sudden and severe deterioration in condition, we called a rather short (1–2 months) phase of illness, in which the patients showed a remarkable enlargement of liver metastases and/or increase in number of tumor lesions along with an increase in transaminases and circulating tumor markers such as chromograninA and/or NSE. We found that a subset of these patients exhibited G3NETs, most of which originated from the pancreas, that had developed NEC-like features, including *TP53* mutations. However, no transformation to a typical NEC was observed.

**Fig. 5:**
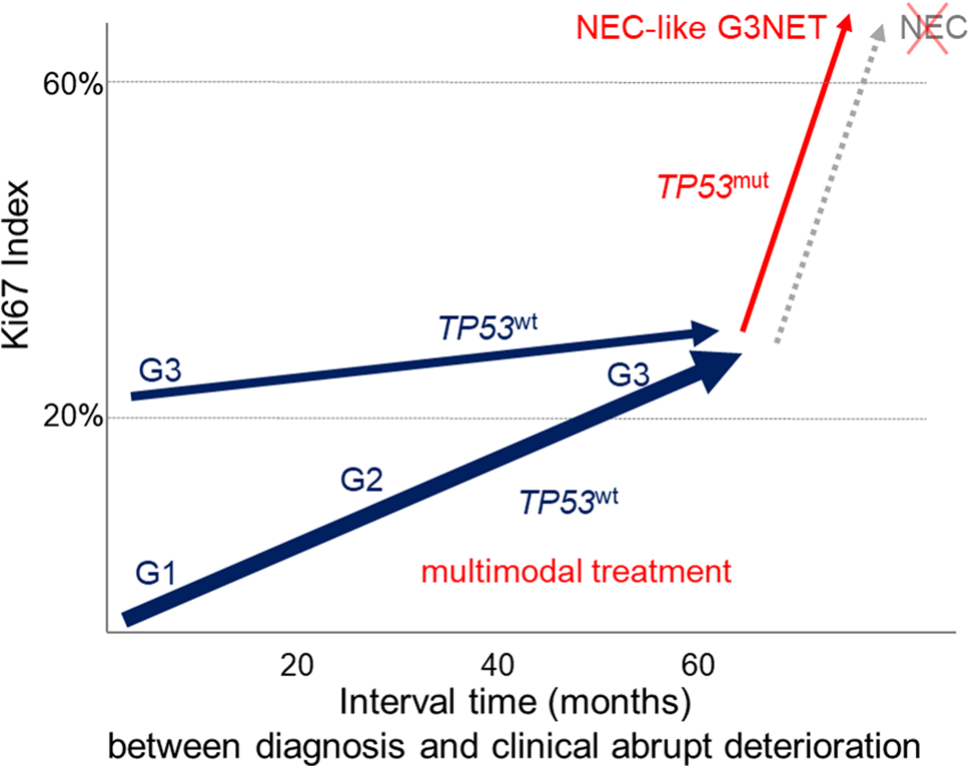
The graph illustrates longitudinal trajectories of progressing metastasized G3 neuroendocrine tumors (G3NETs) under systemic therapies. The majority of G3NETs (28/40, 70%) evolved from G1/G2NETs, while only 30% were primary G3NETs (blue trajectories). After a median interval of 24 months, the delta Ki67 reached a value of 19%. Nine of the 40 G3NETs (22%) exhibited an abrupt deterioration after a median interval time of 60 months, accompanied by NEC-like transformation and a *TP53* mutation (red trajectories). No NEC transformation occurred

In a cohort of 62 NET patients who underwent a tissue examination at the time of diagnosis and subsequently throughout the course of the disease, approximately two-thirds of patients were found to have a G3NET at the last biopsy (40/62, 65%). The G3NETs were observed to emerge from G1NETs (*n* = 4) or G2NETs (*n* = 24), and only 12 patients exhibited a G3NET at the initial examination. Among the G3NET patients, a sudden and severe deterioration in condition was observed in nine patients. In all of these patients, the metastatic tumor tissue exhibited histological changes and immunohistochemical overexpression of p53, accompanied by a corresponding mutation in *the TP53* gene. In eight out of nine NEC-like G3NETs, the allele frequency was high, suggesting monoclonality of the mutation. This somewhat unexpected finding may be explained by the fact that most of the debulking sample contained NEC-like G3NET tissue and was probably the only tissue available for molecular examination. The other G3NETs exhibited no alterations in the *TP53* gene. The NEC-like changes were characterized by high Ki67 values (mean 65, ranging from 50 to 85), a confluent growth pattern, nuclear pleomorphism, and necrosis, which were consistent with large cell NEC features in all but one case, which exhibited characteristics more akin to small cell NEC. The presence of intratumoral heterogeneity [38], with the presence of NET tissue in proximity to NEC-like changes, was a common observation, particularly evident in larger samples. The *TP53* alterations were represented by seven missense mutations with disparate mutation patterns, in addition to a single deletion. *RB1* alterations were observed infrequently, manifesting as mutation and loss of expression in a single case. It is noteworthy that the molecular analysis also identified *MEN1* and *DAXX* mutations in four and two pancreatic NEC-like G3NETs, respectively. As these mutations are characteristic of PanNETs but not NECs, their identification provides compelling evidence that the pancreatic NEC-like G3NETs originated from NETs [3, 5, 39]. Another finding that supports the NET origin of NEC-like G3NETs is the frequent expression of SST2. This has been described in many G3NETs, but rarely in NECs [3]. These findings collectively indicate that despite the presence of advanced histological alterations in conjunction with *TP53* mutations, there is no complete transformation to NECs. We thus designated these neoplasms as “NEC-like G3NET” (Fig. 5). Seven of our nine NEC-like G3NETs originated from the pancreas (mainly from the tail), and two from the rectum. While the pancreas is already known to be the most common site of G3NET origin [1, 8, 30, 40], the rectum is an uncommon site for G3NET [1]. Consequently, detailed data on high-grade rectal NETs are not available. A review of the literature reveals that the only extrapancreatic NETs that have been observed to progress from G1/G2 to G3NET and acquire a *TP53* mutation have been reported in the lung and thymus [28, 29, 41].

Two recent studies conducted on the pancreas have yielded comparable results regarding the development of high-grade pancreatic neoplasms, aligning with the findings of our investigation [26, 30]. Umetsu et al. employed an integrated molecular and immunohistochemical approach to examine 47 high-grade pancreatic neoplasms, identifying 34 high-grade PanNENs, either as G3NETs or NECs, after excluding 17 mixed acinar NECs [26]. As expected, PanNECs frequently exhibited alterations in *TP53*,* RB1*, and *CDKN2A*, but also in *KRAS* and *SMAD4*. In contrast, G3PanNETs harbored NET-typical gene alterations, including *MEN1*, *DAXX*,* ATRX*,* TSC2*,* SETD2*, and *CDKN2A*. Furthermore, 35% (6/17) of these NETs exhibited *TP53* alterations, which are comparable to our NEC-like G3NETs [26]. The resemblance of Umetsu’s TP53-mutated G3PanNETs to our NEC-like G3NETs is further substantiated by the observation that their Ki67 values were markedly elevated in comparison to those observed in G3PanNETs lacking *TP53* mutation.

The other investigation is a longitudinal study that reported a transformation from NET to NEC in seven out of 152 patients with gastroenteropancreatic or pulmonary NETs after PRRT treatment [30]. Molecular analysis of the five high-grade NENs, all of which originated from the pancreas, revealed the presence of *TP53* mutations in three of the five cases [30]. Given that the *TP53* mutations were partially accompanied by other gene alterations, including *ATRX*,* TSC2*,* TSC1*, and *PIK3C*A, which have been previously reported in PanNETs [42], it can be inferred that these high-grade PanNENs also belong to the group designated as NEC-like G3NETs. Cordero-Hernandez designated the tumors as NECs, yet a detailed description is absent, thereby rendering it conceivable that the tumors’ histology aligns with ours. Additionally, high-grade transformation with *TP53* mutation has been documented in two other PanNETs during tumor progression [27, 43]. The aforementioned studies, including our own, collectively indicate that* TP53* mutations that emerge following multimodal treatment may facilitate the evolution of NETs into high-grade NENs. It appears that *RB1* does not exert a significant influence on this phenomenon, as evidenced by the observation of a single *RB1* alteration in our series of NEC-like G3NETs.

All studies of NET progression, with the exception of one whose study design and complex results are challenging to interpret [31] and compare with other investigations, concur that the majority of tumors evolve from low-grade to high-grade NETs. However, there is a paucity of knowledge regarding the time frame for NET progression, as this is difficult to ascertain due to the definition of the endpoint. Additionally, the endpoint of the interval time could not be precisely defined in our study, as it was dependent on the decision of the patient’s physician regarding the timing and necessity of a biopsy. Nevertheless, the presence of analogous alterations in all endpoint biopsies led to the conclusion that, at the time of biopsy, the transformation process was still ongoing in all patients. Accepting this somewhat imprecise endpoint determination, the interval time between diagnosis and rapidclinical deterioration with concomitant NEC-like histology was unexpectedly much longer with a median interval time of 60 months (range 30–168, with the longest interval time in the two rectal NEC-like G3NETs) than the interval time of 24 months (range 7–180) in G3NET patients without abrupt disease deterioration (Fig. 1). A notable distinction was observed in the Ki67 delta values at the endpoint of the interval time, with a median delta Ki67 value of 53% (range 37 to 68) in NEC-like G3NETs, in contrast to a median delta Ki67 value of 19% in G3NETs without NEC-like features (Fig. 1). Our findings are generally consistent with those of Cordero-Hernandez, who reported a median interval time between diagnosis and transformation to high-grade NEN of 83 months (range 17–210) and Ki67 values up to 90% (range 51 to > 90%) at the endpoint of the interval time [30]. These observations indicate that the *TP53* mutation, which is likely to be a primary driver of the accelerated growth observed in one or more of the metastases, is frequently a relatively late event in G3NETs undergoing systemic treatment.

The current standard of care for G3NETs is not yet established and is currently being investigated in clinical trials. These include the use of PRRT, chemotherapy with either CAPTEM (capecitabine and temozolomide) or FOLFOX (folinic acid fluorouracil and oxaliplatin), and everolimus [44]. As demonstrated in the aforementioned study by Cordero-Hernandez, seven patients who exhibited abrupt progression demonstrated a high-grade transformation subsequent to PRRT following temozolomide chemotherapy [30]. In the present study, three of the nine patients with NEC-like G3NETs who received PRRT prior to the final examination were examined for tumor mutational burden, microsatellite instability, and PD-L1 status. No differences were identified compared to the findings in G3NETs without NEC-like transformation. Although the question regarding a potential correlation between treatment and accelerated disease progression is of significant interest, it is beyond the scope of this investigation due to the limited number of NEC-like G3NET patients, which is insufficient to yield meaningful insights.

In conclusion, a retrospective longitudinal study comprising baseline and follow-up biopsies identified the development of G3NETs with NEC-like changes in a cohort of 40 G3NET patients. Of these, nine patients exhibited rapid progression in liver metastases from seven pancreatic NETs and two rectal NETs. It is reasonable to conclude that this phenomenon is attributable to the novel *TP53* mutation, which was detected in all nine cases at the last evaluation, and which likely accelerated tumor growth and altered tumor structure. Additionally, our findings indicated that a considerable number of pancreatic NEC-like G3NETs exhibited mutation characteristic of PanNETs, including *MEN1* and *DAXX*. This observation lends support to the hypothesis that no morphomolecular transformation into a typical NEC had occurred. Future studies with spatial transcriptomics may be helpful, to determine the area in the G3NET and NEC-like G3NET where the genetic transformation occurs.

## Supplementary Information

Below is the link to the electronic supplementary material.

ESM 1 (TIF 67.9 KB)

ESM 2 (DOCX 17.7 KB)

ESM 3 (DOCX 16.8 KB)

ESM 4 (DOCX 18.8 KB)

ESM 5 (DOCX 16.9 KB)

## Data Availability

The Datasets used and analyzed during the current study are available from the corresponding author on reasonable request.
