# Supplementary figures and images for: Rapid Evolution of Metastases in Patients with Treated G3 Neuroendocrine Tumors Associated with NEC-Like Transformation and TP53 Mutation
Source: Endocr Pathol. 2024 Oct 9;35(4):313–24. doi: 10.1007/s12022-024-09827-y (PMC11659366; doi:10.1007/s12022-024-09827-y)

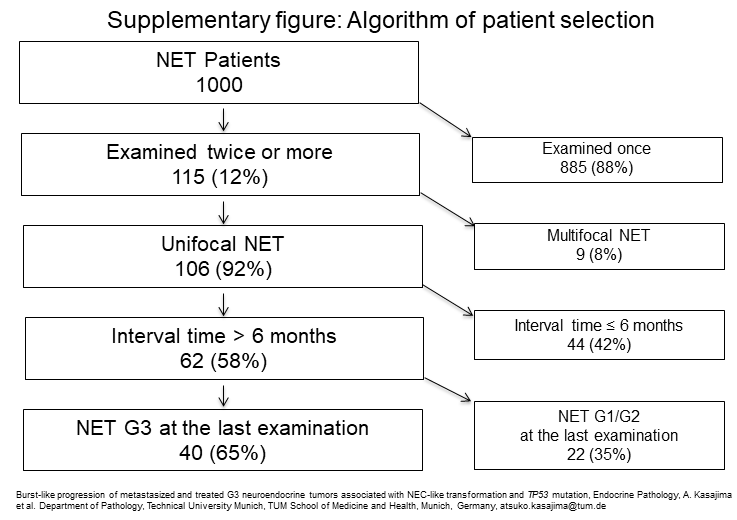

Supplement: Supplementary file 1 — (TIF 67.9 KB) [file 12022_2024_9827_MOESM1_ESM.tif]
