# Supplementary material for: Rapid Evolution of Metastases in Patients with Treated G3 Neuroendocrine Tumors Associated with NEC-Like Transformation and TP53 Mutation
Source: Endocr Pathol. 2024 Oct 9;35(4):313–24. doi: 10.1007/s12022-024-09827-y (PMC11659366; doi:10.1007/s12022-024-09827-y)
Supplement: Supplementary file 2 — (DOCX 17.7 KB) [file 12022_2024_9827_MOESM2_ESM.docx]

| Supplementary Table 1. Clinicopathological data in 62 patients with neuroendocrine tumor examined twice | | | |
| --- | --- | --- | --- |
|  |  | N | % |
| Total |  | 62 | 100 |
| Age | Median (range) | 57 (23-81) | - |
| Sex | Male : Female | 34 : 28 | 55 : 45 |
| Primary Organ |  |  |  |
|  | Pancreas | 31 | 50 |
|  | Lung/Thymus | 15 | 24 |
|  | Ileum | 7 | 11 |
|  | Rectum | 3 | 5 |
|  | Others^a^ | 6 | 10 |
| Functionality |  |  |  |
|  | Non-functional | 54 | 87 |
|  | Functional^b^ | 8 | 13 |
| Hereditary diease |  |  |  |
|  | none | 61 | 98 |
|  | NF1 | 1 | 2 |
| Material status |  |  |  |
| in first examination | Biopsy | 27 | 44 |
|  | Resection | 35 | 56 |
| in last examination | |  |  |
|  | Biopsy | 52 | 84 |
|  | Resection | 10 | 16 |
| Indication for second assesments | |  |  |
|  | Review of diagnosis | 52 | 84 |
|  | Primary resection | 2 | 3 |
|  | Tumor reduction | 8 | 13 |
| delta Ki67 (difference initial and last assessment) | | |  |
|  | Median (range) | 16 (1-68) |  |
| Interval time (months) | |  |  |
|  | Median (range) | 29 (6 - 180) |  |
| WHO Grade at the last examination | |  |  |
|  | G1/G2 | 22 | 35 |
|  | G3 | 40 | 65 |
| WHO Grade (first - last assessment) | |  |  |
|  | G1 - G1 | 1 | 2 |
|  | G1 - G2 | 12 | 19 |
|  | G2 - G2 | 9 | 15 |
|  | G1 - G3 | 4 | 6 |
|  | G2 - G3 | 24 | 39 |
|  | G3 - G3 | 12 | 19 |
| Therapy between the initial and last examination ^d^ | | |  |
|  | None | 12 | 25 |
|  | Performed | 36 | 75 |
| Abbreviations: NET neuroendocrine tumor, a) 2 renal, 1 gastric (type 3), duodenal, breast, presacral NET each, b) 1 patient with glucagonoma, carcinoid syndrome and insulinoma each, 2 patients with Zollinger Ellison syndrom and Cushing syndrome each, 1 patient with Zollinger-Ellison and Insulinoma, 6) data missing in 14 patients | | | |

Burst-like progression of metastasized and treated G3 neuroendocrine tumors associated with NEC-like transformation and *TP53* mutation, Endocrine Pathology, A. Kasajima et al. Department of Pathology, Technical University Munich, TUM School of Medicine and Health, Munich, Germany, atsuko.kasajima@tum.de
