# Supplementary material for: Rapid Evolution of Metastases in Patients with Treated G3 Neuroendocrine Tumors Associated with NEC-Like Transformation and TP53 Mutation
Source: Endocr Pathol. 2024 Oct 9;35(4):313–24. doi: 10.1007/s12022-024-09827-y (PMC11659366; doi:10.1007/s12022-024-09827-y)
Supplement: Supplementary file 3 — (DOCX 16.8 KB) [file 12022_2024_9827_MOESM3_ESM.docx]

| Supplementary Table 2. Detailed information of immunohistochemical stainings | | | | |
| --- | --- | --- | --- | --- |
| Primary antibody | Source | Clone/product number | Host | Dilution |
| Synaptophysin | Ventana/Roche, Arizona, USA | MQR40 | Rabbit, monoclonal | 1:1 |
| Chromogranin A | ThemoFisher, Waltham,USA | LK2H10 | Mouse, monoclonal | 1:500 |
| Ki67 | Dako, Glostrup, Denmark | MIB1 | Mouse, monoclonal | 1:50 |
| p53 | Dako, Glostrup, Denmark | DO-7 | Mouse, monoclonal | 1:200 |
| Retinoblasoma1 | BD Biosciences, Heidelberg, Germany | G3-245 | Mouse, monoclonal | 1:200 |
| SST2A | Zytomed Systems, Berlin, Germany | RBK 046-05 | Rabbit, polyclonal | 1:100 |
| Islet1 | Abcam, Cambridge, UK | ab86472 | Mouse, monoclonal | 1:500 |
| CDX2 | Abcam, Cambridge, UK | ab76541 | Rabbit, monoclonal | 1:500 |
| ARX | Merck Millipore, Billerica, USA | 11F6.2 | mouse monoclonal | 1:2000 |
| PDX1 | Abcam, Cambridge, UK | EPR3358(2) | rabbit recombinant monoclonal | 1:2000 |
| Glucagon | Cell Marque, California, USA | 259A | Rabbit, polyclonal | 1:100 |
| Somatostatin | Dako, Glostrup, Denmark | A0566 | Rabbit, polyclonal | 1:1000 |
| PP | Sigma-Aldrich, St. Louis, USA | HPA032122 | Rabbit, polyclonal | 1:2000 |
| Insulin | LSBio, Shirley, USA | HB125 | Rabbit, Polyclonal | 1:1000 |
| Serotonin | Dako, Glostrup, Denmark | 5HT-H209/M075801-2 | Mouse, monoclonal | 1:100 |
| ACTH | Dako, Glostrup, Denmark | 02A3 | Mouse, monoclonal | 1:2000 |
| Gastrin | Dako, Glostrup, Denmark | A56801-2 | Rabbit, polyclonal | 1:3000 |
| Calcitonin | Leica Biosystems, Nußloch, Germany | CL1948 | Mouse, monoclonal | 1:600 |
| p16 | Ventana/Roche, Arizona, USA | E6H4 | Mouse, monoclonal | 1:1 |
| Footnote: Abbreviations: SST2A somatostatin receptor type 2A | | | | |

Burst-like progression of metastasized and treated G3 neuroendocrine tumors associated with NEC-like transformation and *TP53* mutation, Endocrine Pathology, A. Kasajima et al. Department of Pathology, Technical University Munich, TUM School of Medicine and Health, Munich, Germany, atsuko.kasajima@tum.de
