# Supplementary material for: Rapid Evolution of Metastases in Patients with Treated G3 Neuroendocrine Tumors Associated with NEC-Like Transformation and TP53 Mutation
Source: Endocr Pathol. 2024 Oct 9;35(4):313–24. doi: 10.1007/s12022-024-09827-y (PMC11659366; doi:10.1007/s12022-024-09827-y)
Supplement: Supplementary file 4 — (DOCX 18.8 KB) [file 12022_2024_9827_MOESM4_ESM.docx]

| Supplementary Table 3. Molecular genetic data of 12 pancreatic G3 neuroendocrine tumors (G3NET) with and without NEC-like features. | | | | |
| --- | --- | --- | --- | --- |
|  |  | Pancreatic G3NET | | p-value |
|  |  | without  NEC-like features | with  NEC-like feature |  |
|  | Total N (%) | 13 (65) | 7 (35) |  |
| with molecular data | Total N (%) | 5 (42) | 7 (58) |  |
| *TP53* | wt | 5 (100) | 0 | < 0.0001 |
|  | mut | 0 | 7 (100) |  |
|  |  |  | c.413C>A |  |
|  |  |  | c.832C>A |  |
|  |  |  | c.824G>T |  |
|  |  |  | c.830G>T |  |
|  |  |  | c.772G>A |  |
|  |  |  | c.331_332del |  |
|  |  |  | c.1146del |  |
| *RB1* | wt | 5 (100) | 7 (100) | NS |
|  | mut | 0 | 0 |  |
| *MEN1* | wt | 3 (60) | 3 (43) | NS |
|  | mut | 2 (40) | 4 (57) |  |
|  |  | c.1365+1G>T | c.1525delC |  |
|  |  | c.793C>T | c.974C>T |  |
|  |  |  | c.1200+2del |  |
|  |  |  | c.1594C>T |  |
| *DAXX* | wt | 2 (40) | 5 (71) | NS |
|  | mut | 3 (60) | 2 (29) |  |
|  |  | c.1A>G | c.208-1G>T |  |
|  |  | c.340del | c1560_1561del |  |
|  |  | c.1985del |  |  |
| *ATRX* | wt | 5 (100) | 7 (100) | NS |
|  | mut | 0 | 0 |  |
| TMB |  |  |  |  |
|  | median mut/mb (range) | 3.9 (0.8-248) | 11.9 (0.8 - 357) | NS |
|  | Low (<5) | 3 | 1 | NS (0.08) |
|  | Intermediate (5-15) | 0 | 4 |  |
|  | High (>15) | 2 | 2 |  |
| Instable microsatellites (%) | |  |  |  |
|  | median % (range) | 4 (1-4) | 2 (0-4) |  |
|  | MSS | 7 (88) | 7 (100) | NS |
|  | MSI intermediate | 1 (13) | 0 |  |
|  | MSI high | 0 | 0 |  |
| PD-L1 |  |  |  |  |
|  | TPS | 0 (0-5) | 0 (0-5) | NS |
|  | IC | 0 (0-3) | 1 (0-10) |  |
|  | CPS | 0.5 (0-8) | 0.5 (0-7) |  |
| Footnote: Abbreviation: TMB tumor mutation burden, wt wild-type, NS not statistically significant, MSS microsatelite stable, MSI microsatelite instable | | | | |

Burst-like progression of metastasized and treated G3 neuroendocrine tumors associated with NEC-like transformation and *TP53* mutation, Endocrine Pathology, A. Kasajima et al. Department of Pathology, Technical University Munich, TUM School of Medicine and Health, Munich, Germany, atsuko.kasajima@tum.de
