# Supplementary material for: Rapid Evolution of Metastases in Patients with Treated G3 Neuroendocrine Tumors Associated with NEC-Like Transformation and TP53 Mutation
Source: Endocr Pathol. 2024 Oct 9;35(4):313–24. doi: 10.1007/s12022-024-09827-y (PMC11659366; doi:10.1007/s12022-024-09827-y)
Supplement: Supplementary file 5 — (DOCX 16.9 KB) [file 12022_2024_9827_MOESM5_ESM.docx]

| Supplementary Table 4: Treatment modules of patients with G3 neuroendocrine tumors (G3NETs) with and without NEC-like features | | | | | | |
| --- | --- | --- | --- | --- | --- | --- |
|  |  |  |  | G3NET | |  |
|  |  |  |  | without  NEC-like features | with  NEC-like features |  |
| Total N (%) |  |  | 40 (100) | 31 (78) | 9 (22) |  |
| Total patients with treatment data available | | | 31 (100) | 23 (74) | 8 (26) |  |
| Treatment between initial and last examination | | |  |  |  |  |
|  |  | none | 3 (10) | 3 (13) | 0 | NS |
|  |  | at least with one module | 28 (90) | 20 (87) | 8 (100) |  |
|  |  | one module | 9 | 8 | 1 | 0.037 |
|  |  | two modules | 5 | 5 | 0 |  |
|  |  | three modules | 8 | 6 | 2 |  |
|  |  | four modules | 5 | 2 | 3 |  |
|  |  | more than five modules | 2 | 0 | 2 |  |
| Treatment prior to last examination | | |  |  |  |  |
|  |  | none | 2 (7) | 2 | 0 |  |
|  |  | PRRT | 8 (26) | 5 | 3 | NS |
|  |  | SSA | 2 (7) | 1 | 1 |  |
|  |  | Everolimus | 2 (7) | 1 | 1 |  |
|  |  | Sunitinib | 1 (3) | 0 | 1 |  |
|  |  | Alkylating agents | 14 (45) | 12^a^ | 2^b^ |  |
|  |  | Others | 1 (3) | 1^c^ | 0 |  |
| Footnote: Abbreviations: NEC neuroendocrine carcinoma, PRRT peptide receptor radionuclide therapy, SSA somatostatin analog, a) 6 patients treated with FOLFOX (folinic acid fluorouracil and oxaliplatin), 3 with CAP/TEM (capecitabin and temozolomide) and Carboplatin/Etoposid, 1 with STZ/5FU (streptozotocin and 5-fluorouracil). b) 1 patient treated with FOLFOX, 1 with carboplatin/oxaliplatin. c) hormonal therapy | | | | | | |

Burst-like progression of metastasized and treated G3 neuroendocrine tumors associated with NEC-like transformation and *TP53* mutation, Endocrine Pathology, A. Kasajima et al. Department of Pathology, Technical University Munich, TUM School of Medicine and Health, Munich, Germany, atsuko.kasajima@tum.de
